# Dynamics of peripheral perfusion parameters in elective coronary artery bypass graft patients

**DOI:** 10.1186/cc9497

**Published:** 2011-03-11

**Authors:** M Van Genderen, J Boszhuizen, A Pinto Lima, D Gommers, J Bakker, J Van Bommel

**Affiliations:** 1 Erasmus MC, Rotterdam, the Netherlands

## Introduction

Recent studies have suggested that microvascular perfusion impairment may play a role in the development of postoperative organ dysfunction in patients undergoing high-risk or cardiac surgery [1,2]. Postoperative monitoring of tissue perfusion parameters could therefore be used for early detection of tissue hypoperfusion and serve as an endpoint for resuscitation. For this purpose we measured regional and microvascular perfusion parameters in relation to systemic hemodynamics in patients undergoing open heart surgery.

## Methods

We observed 10 consecutive patients who underwent elective coronary artery bypass grafting with cardiopulmonary bypass during the immediate postoperative resuscitation in the ICU. Tissue perfusion was measured directly after admission and repeated before detubation, and consisted of sublingual SDF imaging, forearm Tskin-diff, finger peripheral perfusion index, finger capillary refill time (CRT) and thenar tissue oxygenation (StO_2_). Cardiac output was measured with NICOM bioreactance.

## Results

CO (4.33 ± 1.63 vs. 5.37 ± 1.29) (*P *< 0.05) and central temperature (35.30 ± 0.24 vs. 36.56 ± 0.13) (*P *< 0.01) increased significantly. All tissue perfusion parameters (that is, SDF parameters (MFI ≥2.5; PPV ≥95%), StO_2 _≥80%, CRT ≥5 seconds, Tskin-diff ≤3 and PFI ≥1.4) were within the normal range at admission and did not change significantly until detubation. Even the central-to-toe temperature difference showed no significant difference or correlation between cardiac output and any other peripheral tissue perfusion parameter. The postoperative course was uncomplicated in all patients.

## Conclusions

In the postoperative period, peripheral and microvascular tissue perfusion parameters are not impaired in our CABG patients. Although from a small population, these data suggest that these parameters are not suitable for routine use as an extra hemodynamic resuscitation endpoint. This is in contrast with previous studies and might be explained by differences in study population or measurement interval.
